# Understanding European Consumers' Perception of Food Safety Risks: A Multicountry Analysis of Raw Milk and Raw Milk‐Based Cheeses

**DOI:** 10.1002/fsn3.70409

**Published:** 2025-06-08

**Authors:** Simone Belluco, Anna Pinto, Giulia Mascarello, Stefania Crovato, Marzia Mancin, Aurora Boscolo Anzoletti, Carmen Losasso

**Affiliations:** ^1^ Istituto Zooprofilattico Sperimentale Delle Venezie Viale dell'Università Legnaro Padua Italy

**Keywords:** food safety, multicountry survey, raw milk, risk perception

## Abstract

Food risk perception involves an individual's assessment of the presence of safety attributes in food. Food risk perception is essential for ensuring food safety, as it significantly shapes consumer attitudes. These attitudes, in turn, influence industrial processes and steer both public and private regulatory frameworks. Therefore, it is crucial for food‐chain producers and regulatory authorities to thoroughly examine the key drivers of this concept. For this reason, a multicountry survey was conducted to gain a comprehensive understanding of European citizens' perceptions of food safety risks and health issues related to the consumption of raw milk and raw milk‐based cheeses, with a particular focus on short agri‐food chains. In this study, a thousand citizens from five European countries (Italy, France, Poland, Germany, and Sweden) participated in an online quantitative social research survey. The survey utilized a structured questionnaire administered via the computer‐assisted web interviewing (CAWI) method. The results highlighted that the consumption of raw milk products is widespread within the EU and the perception about risk is low across the investigated countries. The obtained information provides valuable insights, as a low risk perception is linked to a high consumption pattern; thus, to reduce the health impact of raw milk products, risk managers should plan and conduct communication campaigns aimed at raising consumer awareness about food safety risks.

## Graphical abstract

Food risk perception involves an individual's assessment of the presence of safety attributes in food. The survey utilized a structured questionnaire administered via the computer‐assisted web interviewing (CAWI) method. The results provide valuable insights to inform risk communication strategies to be adopted by risk managers.

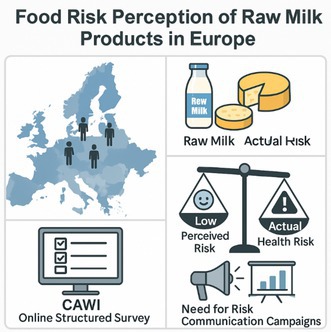

## 1 Introduction

### 1.1 State of the Art

According to EU legislation, “raw milk” is defined as milk produced by the secretion of the mammary gland of farmed animals that has not been heated above 40°C or undergone any treatment with an equivalent effect (Reg. [EC] 853/20044).

Raw milk is associated with potential hazards, including *Listeria monocytogenes* , verocytotoxin‐producing *Escherichia coli* (VTEC), *Staphylococcus aureus* , *Salmonella*, and *Campylobacter* (Verraes et al. 2015; West 2008).

In addition to the microbiological hazards specific to raw milk, there are also chemical hazards that cannot be mitigated through pasteurization and thus are common to both milk and milk products.

Despite these risks, consumer interest in raw milk cheese remains high, driven by its unique flavor and the potential benefits of its natural microbial community (Yoon et al. 2016). The risks and benefits of raw milk consumption are thus debated, with both science‐based and value‐based arguments being presented (West 2008). Risk management practices vary significantly worldwide, reflecting different policy approaches. In Australia, for example, the sale of raw milk without pasteurization is prohibited because of its inherent risk (Linn 2019), whereas in most EU member states, its sale is permitted.

In addition to raw milk, raw milk‐based cheese is also consumed. These constitute a subcategory of specialty cheese characterized by the use of unpasteurized milk in its manufacture. Additionally, this production is often characterized by small‐scale and limited‐volume operations. The interest in this sector is likely due to the increased sophistication of consumers, who seek a greater variety of unique, distinctive, hand‐crafted cheeses. As a result, specialty food stores and farmers' markets have expanded their cheese offerings.

A sociological study of consumer preferences has revealed an increasing market share for specialty cheese, which aligns with the theory of postmodern consumerism (Kupiec and Revell 1998). This consumer approach is characterized by a “hostility toward generalization” where the plurality, diversity, and originality of products create value for a purchaser whose identities are forged through the act of choosing and developing “connoisseur” skills. Raw milk‐based cheese fulfills these expectations by offering a selection of unique and often scarce products, which are manufactured via diverse and often historically or regionally traditional methods. Furthermore, the experience of purchasing artisanal cheese typically involves interactions with specialists at grocery stores, small shopkeepers, or even artisanal producers, thereby encouraging consumers to develop “food literacy” skills.

Raw milk cheeses occupy the high end of the specialty market. Their artisanal production allows for the development of short supply chains, providing a high return to farmers and making small‐scale agriculture profitable. In fact, many small dairies that produce artisanal cheese often also farm milk‐producing animals themselves and are located across the country. Supporting these productions is a viable way to promote local agriculture and maintain regional distinctiveness. Geographic factors, including climate, soil, grass, milk, and the presence of specific animals and vegetation in the cheese production area, are strongly associated with the taste and quality of cheese.

However, despite these products meeting growing consumer demand, concerns regarding their safety for human health have arisen by public authorities in Member States, as foodborne outbreaks related to contaminated raw milk cheese continue to be reported in the European Union (EFSA and ECDC 2023). Long ripening periods and pasteurization are the recommended technologies to mitigate the risk posed by contaminating foodborne pathogens. Nonetheless, it has been demonstrated that *Escherichia coli* O157:H7, *Salmonella*, and *Listeria* can survive a 60‐day aging period (Cosciani‐Cunico et al. 2015). Additionally, pasteurization can impact the original bacterial community, which is essential for the typical flavors and aromas of different raw milk cheeses (Montel et al. 2014).

Previous studies have examined consumers' attitudes toward raw milk and raw milk cheese in individual countries or within specific cultural or economic contexts (e.g., Kühl et al. 2017; Visschers and Siegrist 2015; Colonna et al. 2011). However, there is limited comparative data across multiple EU countries that reflect the diversity of raw milk product traditions and food safety awareness. Additionally, little is known about how perceptions of risk interact with sociodemographic characteristics, trust in institutions, and the degree of familiarity with artisanal foods. This gap limits our understanding of consumer profiles and hinders the development of targeted risk communication strategies.

Surveys are a valuable method for exploring the reasons behind consumer habits. Within the EU, there is a wide range of variations due to different experiences across countries, which are largely influenced by geographical, climatic, and historical factors. When investigating such behaviors, it is crucial to collect information from various countries that represent different habits.

The present study aims to investigate, through a multicountry cross‐sectional survey, the characteristics, risk perceptions, and consumption habits of consumers of raw milk food products, identifying any differences among the five countries considered.

## 2 Materials and Methods

### 2.1 The Project

The survey was conceived within hALO, an EU‐funded research project aimed at implementing advanced photonics technologies to provide a new, multianalytic, highly sensitive, and robust sensor for fast onsite analysis. To increase project impact, a multiactor approach was used to create synergies among researchers, SMEs, end users, and other relevant stakeholders. The survey was the tool chosen to understand consumer risk perception in relevant food supply chains to include their concerns in sensor design and development.

### 2.2 Selection of Countries

Five countries were selected to administer the questionnaire. Selected countries were Italy, Germany, Poland, France, and Sweden. These countries were chosen to maximize the heterogeneity and representativeness of the EU, particularly regarding raw milk products such as cheese.

Italy and France are renowned for their extensive range of PDO (Protected Designation of Origin) cheeses, reflecting a rich heritage of raw milk cheese production. Italy, with its famous Parmigiano Reggiano and Gorgonzola, and France, with iconic cheeses such as Roquefort and Camembert, have long traditions of artisanal cheese‐making that significantly contribute to their cultural and gastronomic identities (European Commission 2023). These traditions are supported by robust regulations and quality control measures that ensure the safety and authenticity of their products.

Owing to its diverse dairy industry, Germany offers a wide range of cheese varieties and has a strong emphasis on both quality and safety in cheese production. German consumers are highly aware of food safety issues, which influence their consumption patterns and preferences.

Poland, with its growing dairy sector, presents a unique perspective due to its transitional economy and evolving food safety standards. The Polish dairy industry is known for traditional products such as Oscypek, a smoked cheese made from raw sheep milk, highlighting the country's rich cultural heritage and diversity in raw milk cheese production.

Sweden, which represents the Nordic region, adds further diversity to the literature with its distinctive dairy products and stringent food safety regulations. Swedish consumers are known for their high standards regarding food quality and safety, and the country's dairy sector is characterized by a commitment to sustainable and ethical production practices.

By including these five countries, this study aims to capture a comprehensive picture of the characteristics, risk perceptions, and consumption habits related to raw milk products across Europe. This diverse selection ensures that the survey reflects a wide range of cultural, climatic, and regulatory contexts, providing valuable insights into the multifaceted nature of raw milk consumption and production in the EU.

### 2.3 The Survey Instrument

A semistructured questionnaire (Corbetta 2003) was designed on the basis of the research team's experience and the objectives of the research project.

The questionnaire, initially drafted in English, was translated into French, German, Italian, Polish, and Swedish. It was pretested by the local project partners to identify and eliminate any unclear or ambiguous questions and to refine its content. A screening question was introduced to identify consumers of raw milk and raw milk‐based cheese. The definition of raw milk provided at the beginning of the questionnaire aimed to prevent possible misunderstandings: “Raw milk means milk produced by the secretion of the mammary gland of farmed animals that has not been heated to more than 40°C or has undergone any treatment that has an equivalent effect.”

The final questionnaire comprises 21 questions, which are primarily closed‐ended with single or multiple choices, covering the following sections: (1) consumers' food risk perceptions, (2) raw milk consumption, (3) consumption of raw milk‐based cheese, and (4) respondents' sociodemographic characteristics. Details of the questions posed to the respondents in these sections are provided in Table S1.

### 2.4 Data Collection

Data were collected between September and October 2022 through the computer‐assisted web interviewing (CAWI) method. An Italian market research and opinion polling company administered the questionnaire across the five countries, distributing it via email to individuals registered in country‐specific panels.

The sample was designed with a minimum effective achieved sample size of 200 interviewees for each selected country. The sample was stratified by gender and age according to the national population, with no other demographic variables considered.

Data collection was conducted anonymously and adhered to the General Data Protection Regulation (EU) 2016/679.

### 2.5 Data Collection and Statistical Analysis

Descriptive statistics were used to summarize the sociodemographic characteristics of the respondents from the five countries: gender, age, area of origin, educational qualification, and occupation.

Before analysis, certain response options were consolidated (see Table S1). One‐way ANOVA with the Bonferroni post hoc correction was conducted to identify significant differences in food risk perceptions among the countries.

Additionally, considering all the countries together, contingency tables and chi‐squared tests were employed to examine the dependency relationships between categorical variables, whereas Kendall's tau test was utilized to assess the correlations between pairs of ordinal variables. In the case of low representativity of class variables, consolidation of response options was performed.

Cronbach's alpha coefficient was used to assess the internal consistency of the dimension “Food risk perception.”

Weekly raw milk consumption was measured by combining the portion of raw milk consumed and the frequency of raw milk consumption. The amount of raw milk consumed was assessed by asking respondents to indicate the typical portion size of raw milk they habitually consume, selecting one of the response options listed in Table S1. For the measurement of weekly raw milk consumption, the response options were as follows: “150 cc,” “200 cc,” and “250 cc.” For the frequency of raw milk consumption, the response options were recorded on a weekly basis, starting by assigning a score of 1 to the response option “often (at least once per week)” and a score of 0.75 to the response option “sometimes (two/three times per month)” and 0.25 to the response option “rarely (once per month or less).” Finally, the option “always (every day)” was assigned a value of 3. The weekly raw milk consumption was calculated by multiplying these two variables. The data were processed via SPSS (Statistical Package for Social Science) software version 25.0.0.1 for Windows (SPSS Inc., Chicago, Illinois). The level of statistical significance was determined at the 5% level (*α* = 0.05).

## 3 Results

### 3.1 The Sample

Among the 1059 survey respondents, 648 (61.2%) reported consuming raw milk food products and were included in the analyses. Of these, 259 (40%) consumed both raw milk and raw milk‐based cheese, 128 (19.7%) consumed only raw milk, and 261 (40.3%) consumed only raw milk‐based cheese. The sociodemographic characteristics of these consumers, broken down by country, are detailed in Table 1.

**TABLE 1:** Sociodemographic characteristics of consumers of raw milk products (% values on *n*_FR_ = 163, *n*_IT_ = 115, *n*_SE_ = 106, *n*_PL_ = 127, *n*_DE_ = 137 and *n*_Total_ = 648).

Characteristics	FR	IT	SE	PL	DE	Total
Gender						
Female	58.3	45.2	51.9	42.5	56.2	51.4
Male	41.7	54.8	47.2	57.5	43.8	48.4
Other			0.9			0.2
Age (in classes)						
18–29	27.0	19.1	34.9	23.6	29.2	26.7
30–44	31.9	22.6	28.3	41.7	31.4	31.5
45–54	16.0	24.4	17.0	18.9	15.3	18.0
55–64	16.0	20.0	11.3	11.8	13.9	14.7
65–75	9.2	13.9	8.5	4.0	10.2	9.1
Area of origin						
Urban area	52.2	58.3	59.4	79.5	54.7	60.3
Suburban area	18.4	22.6	18.9	14.2	19.0	18.5
Rural area	29.4	19.1	21.7	6.3	26.3	21.2
Educational qualification						
Primary/lower secondary school	26.0	10.4	9.4	0.8	6.6	9.0
Professional qualification	20.9	7.0	15.1	14.2	52.6	22.8
Higher secondary school diploma	32.5	44.3	47.2	44.1	25.5	37.8
University diploma/Degree	29.4	31.3	27.4	34.6	15.3	27.5
Postgraduate	1.2	7.0	0.9	6.3		2.9
Occupation						
Student	8.6	13.1	15.1	3.9	7.3	9.3
Employed	67.5	53.9	65.1	80.3	79.5	69.8
Unemployed	11.0	16.5	9.4	9.5	7.3	10.6
Retired	12.9	16.5	10.4	6.3	5.9	10.3

### 3.2 Food Risk Perception

Risk perception and the consumption of raw milk and raw milk‐based cheeses are closely linked, with distinct differences observed across the five European countries (Table 2). As shown in Table S1, three questions on a Likert scale from 1 to 5 were asked to investigate respondents' perceptions of food risk. The internal consistency of this dimension was *α* = 0.843. One‐way analysis of variance (ANOVA) revealed statistically significant differences between countries regarding the perceived level of chemical hazards in food (*p* = 0.000) and the perceived level of biological hazards (*p* = 0.009). The difference in the general perception of food risk between countries was nearly statistically significant (*p* = 0.050).

**TABLE 2:** One‐way analysis of variance (*n*_FR_ = 163, *n*_IT_ = 115, *n*_SE_ = 106, *n*_PL_ = 127, *n*_DE_ = 137 and *n*_Total_ = 648).

Questions	Countries	Mean	Standard deviation	*F* test	*p*
How exposed do you feel to food risk in general? *Likert scale 1–5*	FR	2.85	1.151	2.388	0.050
	IT	3.02	0.973		
	SE	2.59	1.119		
	PL	2.69	1.193		
	DE	2.77	1.150		
	Total	2.79	1.130		
How exposed do you feel to chemical hazards in food? *Likert scale 1–5*	FR	2.90	1.184	5.968	0.000
	IT	2.96	1.055		
	SE	2.64	1.131		
	PL	3.34	1.063		
	DE	3.04	1.153		
	Total	2.98	1.141		
How exposed do you feel to biological hazards in food? *Likert scale 1–5*	FR	2.96	1.167	3.415	0.009
	IT	3.10	0.949		
	SE	2.65	1.104		
	PL	3.13	1.062		
	DE	2.91	1.134		
	Total	2.96	1.101		

The Bonferroni post hoc correction indicated that Polish respondents perceived chemical hazards significantly more than Swedes (*p* = 0.000) and France (*p* = 0.009) did. Additionally, Swedes had a lower perception of biological hazards than Italians did (*p* = 0.022) and Poles did (*p* = 0.010).

### 3.3 Raw Milk Consumption and Risk Perception

The response options for raw milk consumption were grouped into two categories: “rarely sometimes” (two/three times per month or less) and “often always” (once per week or more) (Table S1). Among the 387 respondents who reported consuming raw milk, 59.9% indicated that they consumed it rarely or sometimes, whereas 40.1% consumed it often or always. Raw milk consumption was most common in France and least common in Sweden. A statistically significant dependent relationship was found between the frequency of raw milk consumption and the country of the respondent (*χ* ^2^ = 11.623, *p* = 0.020), indicating that consumption patterns vary by country (Table 3).

**TABLE 3:** Consumption of raw milk (*n*_FR_ = 68, *n*_IT_ = 61, *n*_SE_ = 56, *n*_PL_ = 99, *n*_DE_ = 103 and *n*_Total_ = 387).

Characteristics	FR	IT	SE	PL	DE	Total
Frequency of consumption						
Rarely sometimes	47.1	57.4	76.8	61.6	59.2	59.9
Often‐always	52.9	42.6	23.2	38.4	40.8	40.1
Portion usually consumed						
Less than a cup (< 200 cc)	38.2	31.1	44.6	29.3	35.9	35.1
A cup (approximately 200 cc)	51.5	65.6	44.6	47.5	49.5	51.2
More than a cup (> 200 cc)	10.3	3.3	10.8	23.2	14.6	13.7

Considering the entire sample, respondents generally reported a relatively low perception of health risk associated with raw milk, with an average score of 2.16 on a Likert scale from 1 to 5. No significant differences in risk perception were observed among the five countries (meanFR = 2.28, meanIT = 2.38, meanSE = 2.23, meanPL = 2.04, meanDE = 2.03; *F* test = 1.894, *p* = 0.111).

The majority of respondents (51.2%) reported drinking approximately 200 cc of raw milk per portion (Table 3). A dependent relationship emerged between the portion of raw milk usually consumed and the country of the respondent (*χ* ^2^ = 18.545, *p* = 0.017). Additionally, considering the entire sample, a statistically significant positive correlation was found between the frequency of raw milk consumption and the quantity of the portion consumed (Kendall's tau = 0.149, *p* = 0.001).

The weekly raw milk consumption, which combines frequency and quantity, ranged from a minimum of 37.5 cc, where a portion of 150 cc is consumed rarely, to a maximum of 750 cc, where a portion of 250 cc is consumed every day. The analysis of variance did not reveal significant differences in weekly raw milk consumption among the countries studied (meanFR = 195.8 cc, meanIT = 225.0 cc, meanSE = 135.3 cc, meanPL = 173.6 cc, meanDE = 177.8 cc, *F* test = 2.371, *p* = 0.052).

### 3.4 Raw Milk‐Based Cheese Consumption and Risk Perception

Among the 520 respondents who reported consuming raw milk‐based cheese, approximately 66% indicated that they did so rarely or sometimes (Table 4). The consumption of raw milk‐based cheese was highest in France and lowest in Sweden and Poland (Table 4). A significant association was found between the frequency of consuming raw milk‐based cheese and the respondents' country of origin (*χ* ^2^ = 26.764, *p* = 0.000), indicating that consumption patterns vary by country.

**TABLE 4:** Consumption of raw milk‐based cheese (*n*_FR_ = 153, *n*_IT_ = 104, *n*_SE_ = 81, *n*_PL_ = 80, *n*_DE_ = 102, and *n*_Total_ = 520).

Characteristics	FR	IT	SE	PL	DE	Total
*Frequency of consumption*						
Rarely sometimes	50.3	67.3	77.8	77.5	68.6	65.8
Often‐always	49.7	32.7	22.2	22.5	31.4	34.2
*Portion consumed per type of cheese*						
Seasoned cheese^a^						
< 50 g	34.0	29.6	27.6	20.0	27.6	28.4
Approximately 50 g	41.5	38.0	37.9	62.9	60.3	47.6
> 50 g	18.9	25.4	27.6	17.1	8.6	19.1
I don't know	5.6	7.0	6.9	0.0	3.5	4.9
Fresh cheese^b^						
< 100 g	44.1	38.2	42.9	26.8	44.4	40.1
Approximately 100 g	40.9	46.1	34.9	50.0	42.6	42.6
> 100 g	7.9	11.8	15.9	21.4	11.1	12.5
I don't know	7.1	3.9	6.3	1.8	1.9	4.8

^a^ *n*_FR_ = 53, *n*_IT_ = 71, *n*_SE_ = 29, *n*_PL_ = 35, *n*_DE_ = 58, *n*_Total_ = 246.

^b^ *n*_FR_ = 153, *n*_IT_ = 104, *n*_SE_ = 81, *n*_PL_ = 80, *n*_DE_ = 102, *n*_Total_ = 520.

The perceived risk associated with raw milk‐based cheese consumption was relatively low, with an average score of 2.14 on a Likert scale from 1 to 5, and there were no significant differences in risk perception among the countries (meanFR = 2.10, meanIT = 2.32, meanSE = 2.27, meanPL = 2.04, meanDE = 2.00, *F* test =2.214, *p* = 0.066).

Regarding the type of cheese consumed, 27 respondents reported not knowing the type of cheese consumed. Among the remaining 493 respondents, 246 (49.9%) indicated that they consumed seasoned cheese, whereas 376 (76.3%) consumed fresh cheese (with the option to select both). In all five countries, most respondents reported consuming approximately 50 g of seasoned cheese per portion (Table 4). For fresh cheese, respondents in Italy and Poland predominantly reported consuming approximately 100 g, whereas in France, Sweden, and Germany, the majority consumed less than 100 g.

**FIGURE 1:**
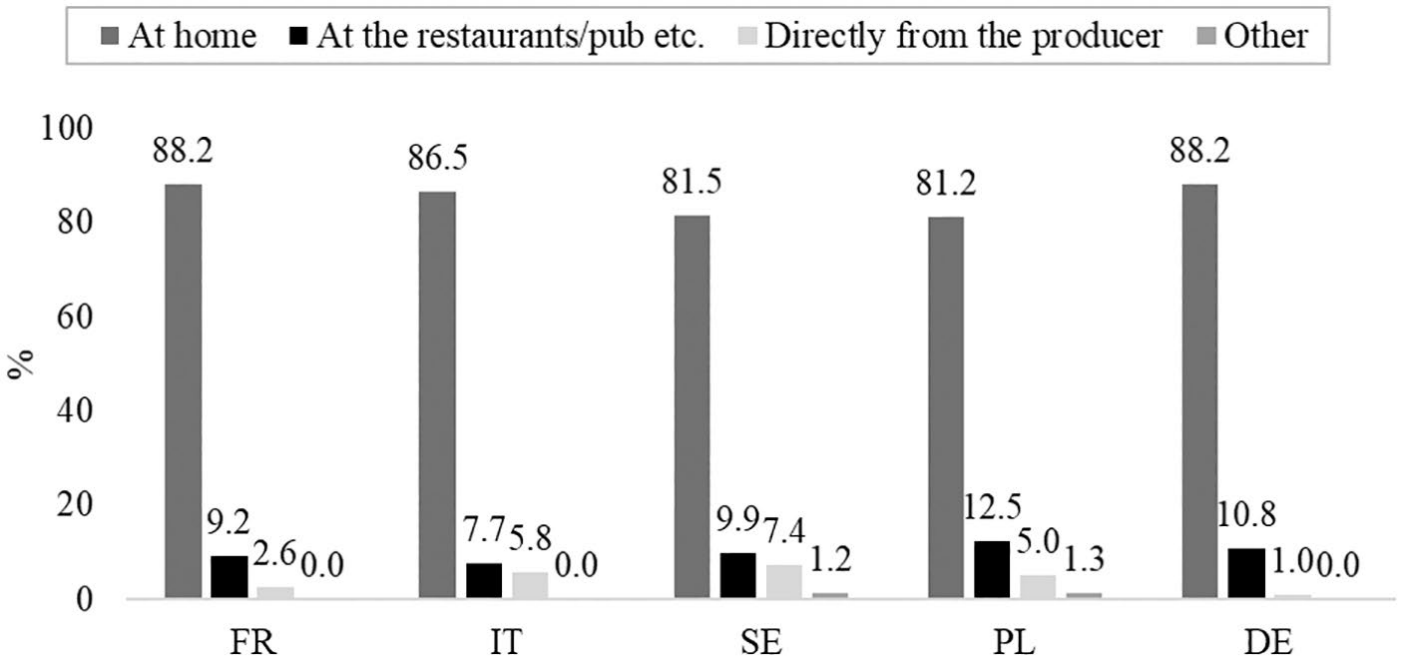
Place where raw milk‐based cheese is usually consumed (*n*_Total_ = 520: *n*_FR_ = 153, *n*_IT_ = 104, *n*_SE_ = 81, *n*_PL_ = 80, *n*_DE_ = 102).

**FIGURE 2:**
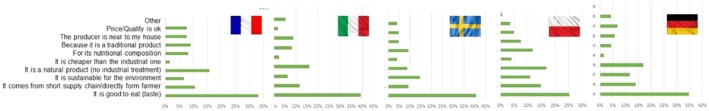
Reasons for raw milk‐based cheese consumption (*n*_Total_ = 520: *n*_FR_ = 153, *n*_IT_ = 104, *n*_SE_ = 81, *n*_PL_ = 80, *n*_DE_ = 102).

Consistent with the findings for raw milk, respondents typically consumed raw milk‐based cheese at home, regardless of their country of origin (Figure 1). When asked about their reasons for consuming raw milk‐based cheese, the primary factor cited was “taste” across all five countries (Figure 2).

## 4 Discussion

The links between raw milk consumption and human illnesses have been clearly demonstrated. The main hazards are *Campylobacter* spp., *Salmonella* spp., shiga toxin‐producing *Escherichia coli* (STEC), *Brucella melitensis* , *Mycobacterium bovis* , and tick‐borne encephalitis virus (EFSA Panel on Biological Hazards (BIOHAZ) 2015). Milk consumption is estimated to cause 4% of all foodborne disease (Grace et al. 2020). In High‐Income Countries, dairy is responsible for around 1% to 6% of reported outbreaks (Claeys et al. 2013). In the US, it has been estimated that unpasteurized milk and derived products, while consumed by only 3.2% and 1.6% of the population, respectively, caused 96% of illnesses attributed to dairy products. This can be quantified in 840 (95% CI 611–1158) times more illnesses and 45 (95% CI 34–59) times more hospitalizations than pasteurized products (Costard et al. 2017). Despite these risks, the consumption of raw milk and derived products is allowed and practiced in most EU countries.

The data collected in this survey are useful within this debate as it deepens into the habits and risk perception at EU level.

Overall, the results indicate that risk perception is low at the EU level. Although significant differences in consumption behaviors were observed between countries, the perception of risk of raw milk products remained consistently low across all groups. This suggests that factors such as taste, product quality, and cultural influences may play a more substantial role in shaping consumer behavior than does risk perception.

Other studies support these observations. For example, a study on Irish farming families reported a common belief that raw milk is risk‐free on the basis of routine test results and is perceived to be of better quality than pasteurized milk (Hegarty et al. 2002).

With respect to quality, the scientific evidence is mixed. While heating raw milk is generally not considered to significantly alter its nutritional value or associated benefits (Claeys et al. 2013), raw milk cheeses are known for their more intense and rich flavor compared with cheeses made with pasteurized milk, largely due to the presence of a diverse native microbiota not found in cheeses made from pasteurized or microfiltered milk (Montel et al. 2014).

In the present study, taste was identified as the primary reason for consuming both raw milk and raw milk cheeses, followed by the perception that these products are natural and free from industrial processing. These findings align with other studies indicating that consumers often view raw milk as more natural, healthier, and better tasting than pasteurized milk does (Baars 2019; Markham et al. 2014).

Another significant factor influencing the choice of raw milk products is the perception that they originate from a short supply chain. This preference for short supply chains and direct relationships with producers reflects a contemporary trend, addressing consumer concerns about the complexity and global nature of modern production processes. These aspects have also been highlighted by other studies (Enticott 2003; Hamilton et al. 2021; Markham et al. 2014), which link the value of raw milk products to ethical considerations related to small‐scale farming and rural settings. These considerations include animal welfare, attention to cattle nutrition, and the sustainability of farming practices.

Consumers' perceptions of the nutritional value and health benefits of raw milk have been highlighted in several studies (Baars 2019; Sabău et al. 2023). Some consumers reported perceived improvements in health, immunity, and mood due to raw milk consumption, especially among those in poor health (Baars 2019). However, the benefits of consuming raw milk must be weighed against the associated risks, underscoring the need for strict hygienic procedures during milking and processing, as well as the importance of mitigating risks through appropriate heat treatment or ripening, particularly for vulnerable groups such as infants (EFSA Panel on Biological Hazards [BIOHAZ] 2015; Yoon et al. 2016).

The strength of benefit perception makes consumers disagreeing with government warnings (Markham et al. 2014). In the US, it has been observed that consumers are willing to pay higher prices for products that carry greater risks (Knutson et al. 2010).

Social and psychological studies on food choices reveal that risk perception is a complex mechanism influenced not only by consumers' knowledge levels but also by their personal attitudes, values, and broader cultural and social context (Finucane and Holup 2005; Fischer and Frewer 2008; Hansen et al. 2003). A crucial factor in food choices, especially concerning artisanal, local, and perceived natural foods such as unpasteurized dairy products, is consumers' trust in the production system and in regulatory and safety controls.

A study conducted in the US reported that the consumption of raw milk cheese is driven primarily by ideological beliefs and taste preferences. Providing scientific information about the safety of pasteurization does not significantly alter consumers' preferences for raw products. Additionally, choosing pasteurized cheese was associated with greater trust in food safety regulation, whereas the preference for raw milk cheese was linked to a higher level of trust in vendors and in food sold directly by producers rather than being regulated by the government (Waldman and Kerr 2018).

These results emphasize the need to contextualize the debate on the regulation of raw milk products by considering factors such as consumer trust and the acceptability of risk within the food system. The perceptions and preferences of consumers are deeply rooted in the cultural differences of each country and are shaped by the regulatory context. The importance of a country's culinary culture and exposure to certain traditional products is also highlighted by studies showing that Southern European countries such as France and Italy tend to prefer unpasteurized cheeses, whereas pasteurized cheeses are more commonly favored in Northern Europe (Meunier‐Goddik and Waite‐Cusic 2019).

The opportunity to consume raw milk and raw milk‐based products remains controversial across different countries, with a wide range of legislative approaches ranging from outright bans (e.g., Australia) to permission and even public support in other countries. In Europe, the situation is similarly varied, as the raw milk market is governed by national legislation. For the countries included in this survey, the current situation can be summarized as follows: in Germany, drinking raw milk is permitted, with sales allowed directly from farmers or through local delivery and sometimes even in local retail stores (Baars 2019). In Italy and France, raw milk can be sold at farms or through vending machines located near farms. In Poland, however, the sale of raw milk for drinking is not permitted (EFSA Panel on Biological Hazards [BIOHAZ] 2015). In Sweden, raw milk can also be sold directly on farms but only in limited weekly quantities, with information about potential hazards provided at the point of purchase.

This complex scenario explains why the debate regarding policy options is open and approaches across countries at the international level are different. In particular contexts, such as EU countries investigated in the present survey, policy options must consider factors beyond food safety alone. Understanding consumers' perceptions of raw milk product safety and their consumption preferences is crucial for regulatory agencies and policymakers to develop effective strategies. It has been suggested that an outright ban could drive consumers to black‐market arrangements, thereby increasing food safety risks due to the lack of official health controls and the risks of fraud associated with the clandestine market (Knutson et al. 2010). However, the lack of a clear policy need to be associate with communication campaign aimed at increased risk awareness and educate consumers toward an appropriate consumption pattern excluding Young, Old, Pregnant and Immunocompromised subjects. Communications could be addressed to the general population or specifically to consumers buying these products. In the first case it is important to design appropriately communication tools to overcome rational bounds and gain trust (Sillence et al. 2016). In the second case a recourse to clear and informative label could be chosen. However this approach alone could not be enough as warning labels often struggle to influence consumers' judgments (Purmehdi et al. 2017).

In every case risk communication cannot replace the implementation of public enforced Food safety management Systems able to mitigate the risks and to protect public health, enhancing safety standards.

The methodology applied in the present study has some limitations. The CAWI method may have sampling bias due to the use of consumer panels composed of volunteer people who may have different characteristics than the general population. Furthermore, online panels may have biases due to the limitations of internet access and the use of devices, especially for specific population groups (e.g., elderly people) or in certain areas, even though in Europe nowadays more than 90% of people declare to use it (Eurostat 2024). However, to minimize the impact of these biases, the survey was carried out with the support of a company specialized in marketing research using profiled panels online, and the sample was designed with similar size in each country and stratified by gender and age in relation to the national populations.

## 5 Conclusion

In conclusion, this study sheds new light on the enduring popularity of raw milk and raw milk‐based products across Europe, revealing that despite extensive debates regarding their safety, these products remain deeply rooted in consumer preferences. While the literature has acknowledged factors such as taste, perceived naturalness, and the appeal of local and traditional food sources as motivators, our findings add a deeper layer by quantitatively demonstrating that these cultural and sensory drivers consistently outweigh risk perceptions across different European contexts.

This study's unique contribution lies in its cross‐country analysis, which highlights significant variations in consumption patterns and regulatory approaches among European nations. Unlike previous studies that focused predominantly on specific countries or regions, our research provides a comprehensive European perspective, revealing how cultural and regulatory differences shape consumer behavior toward raw milk and its derivatives. This approach underscores the necessity of tailoring risk communication strategies and policies to the specific cultural and societal contexts of each country.

Rather than advocating for a one‐size‐fits‐all approach or solely restrictive measures, our study calls for the development of nuanced policies that incorporate consumer education, enhanced safety protocols, and targeted risk communication, especially for vulnerable populations. By leveraging the insights gained from understanding the cultural and psychological drivers behind raw milk consumption, policymakers can craft strategies that resonate with consumers, aligning public health objectives with respect to cultural traditions and consumer demand for these valued food products.

This multifaceted approach does more than address safety concerns; it emphasizes the need for a balanced regulatory framework that not only mitigates risks but also supports consumer autonomy and respects the deep cultural significance of raw milk products. The study's findings highlight the importance of bridging the gap between scientific evidence and consumer beliefs, ultimately aiming to foster an informed public dialogue that aligns food safety with cultural heritage.

## Author Contributions

**Simone Belluco:** conceptualization (equal), funding acquisition (equal), project administration (equal), writing – original draft (equal), writing – review and editing (equal). **Anna Pinto:** data curation (equal), software (equal), writing – original draft (equal). **Giulia Mascarello:** conceptualization (equal), methodology (equal), writing – original draft (equal), writing – review and editing (equal). **Stefania Crovato:** conceptualization (equal), methodology (equal), writing – original draft (equal), writing – review and editing (equal). **Aurora Boscolo Anzoletti:** data curation (equal), software (equal). **Marzia Mancin:** data curation (lead), software (equal), writing – original draft (equal), writing – review and editing (equal). **Carmen Losasso:** conceptualization (equal), funding acquisition (equal), methodology (equal), project administration (equal), writing – original draft (equal), writing – review and editing (equal).

## Conflicts of Interest

The authors declare no conflicts of interest.

## Supporting information


**Table S1.** Questions included in the study.

## Data Availability

The data that support the findings of this study are available from the corresponding author upon reasonable request.
