# Supplementary material for: Understanding European Consumers' Perception of Food Safety Risks: A Multicountry Analysis of Raw Milk and Raw Milk‐Based Cheeses
Source: Food Sci Nutr. 2025 Jun 8;13(6):e70409. doi: 10.1002/fsn3.70409 (PMC12146498; doi:10.1002/fsn3.70409)
Supplement: Supplementary file 1 — Table S1. Questions included in the study. [file FSN3-13-e70409-s001.docx]

**Supplementary materials**

Table S1. Questions included in the study

| **Sections** | **Questions** | **Response options** |
| --- | --- | --- |
| Food risk perception | How exposed do you feel to food risk in general? | Likert scale 1-5: 1=‘not at all exposed’, 5=‘very exposed’ |
|  | How exposed do you feel to chemical hazards* in food?  * A chemical hazard is any substance that can cause a health problem when ingested or inhaled. They include toxins, dangerous chemicals, residue of excess chemicals used in processing food products. | Likert scale 1-5: 1=‘not at all exposed’, 5=‘very exposed’ |
|  | How exposed do you feel to biological hazards* in food?  *Microbiological hazard occurs when food becomes contaminated by microorganisms found in the air, food, water, soil, animals and the human body. Given the right conditions, some microorganisms may cause a foodborne illness. | Likert scale 1-5: 1=‘not at all exposed’, 5=‘very exposed’ |
| Screening question | Which of these foods do you consume?  Raw milk (‘*Raw milk’ means milk produced by the secretion of the mammary gland of farmed animals that has not been heated to more than 40 °C or undergone any treatment that has an equivalent effect*)  Raw milk based cheese | Yes; No; I don’t know |
| Raw milk consumption | How frequently do you drink raw milk?^a^ | Rarely (once per month or less); Sometimes (two/three times per month); Often (at least once per week); Always (everyday) |
|  | How much raw milk do you usually drink per portion? | Less than a cup (less than 200 cc); A cup (approximately 200 cc); More than a cup (more than 200 cc) |
|  | Where do you usually drink raw milk? | At home; At the restaurants/pub etc.; Directly from the producer; Other |
|  | Why do you consume raw milk? *(multiple choice)* | It is good to eat (taste); It comes from short supply chain/directly form farmer; It is sustainable for the environment; It is a natural product (no industrial treatment); It is cheaper than the industrial one; For its nutritional composition; Because it is a traditional product; The producer is near to my house; It has a good quality/price ratio; Other |
|  | How risky do you think raw milk is for your health? | Likert scale 1-5: 1=‘not at all risky’, 5=‘very risky’ |
| Raw milk based-cheese consumption | How frequently do you eat raw milk-based cheese?^a^ | Rarely (once per month or less); Sometimes (two/three times per month); Often (at least once per week); Always (everyday) |
|  | What kind of raw milk based-cheese do you consume? *(multiple choice)* | Seasoned cheese; Fresh cheese; I don’t know |
|  | *(If seasoned cheese is consumed)* How much seasoned raw milk based-cheese do you consume per portion? | Less than 50 g; Approximately 50 g; More than 50 g; I don’t know |
|  | *(If fresh cheese is consumed)* How much seasoned raw milk-based cheese do you consume per portion? | Less than 100 g; Approximately 100 g; More than 100 g; I don’t know |
|  | Where do you usually consume raw milk-based cheese? | At home; At the restaurants/pub etc.; Directly from the producer; Other |
|  | Why do you consume raw milk-based cheese? | It is good to eat (taste); It comes from short supply chain/directly form farmer; It is sustainable for the environment; It is a natural product (no industrial treatment); It is cheaper than the industrial one; For its nutritional composition; Because it is a traditional product; The producer is near to my house; It has a good quality/price ratio; Other |
|  | How risky do you think raw milk-based cheese is for your health? | Likert scale 1-5: 1=‘not at all risky’, 5=‘very risky’ |
| Sociodemographic data | Gender | Female; Male; Other |
|  | Age | Open questions |
|  | I live in | Urban area (in a city or town); Suburban area (just outside of a city or town); Rural area (out in the country) |
|  | Educational qualification | Primary/lower secondary school; Professional qualification; Higher secondary school diploma; University diploma/Degree; Postgraduate |
|  | Occupation | Student; Employed; Unemployed; Retired |

^a^The response options were combined into two categories: ‘Rarely sometimes (two/three times per month or less)’ and ‘Often always (once per week or more)’ before analysis.
